# Multi-center feasibility study evaluating recruitment, variability in risk factors and biomarkers for a diet and cancer cohort in India

**DOI:** 10.1186/1471-2458-11-405

**Published:** 2011-05-27

**Authors:** Rashmi Sinha, Carrie R Daniel, Niveditha Devasenapathy, Hemali Shetty, Susan Yurgalevitch, Leah M Ferrucci, Preethi S George, Kerry Grace Morrissey, Lakshmy Ramakrishnan, Barry I Graubard, Kavita Kapur, K Srinath Reddy, Mary J McAdams, Tanuja Rastogi, Nilanjan Chatterjee, Prakash C Gupta, Sholom Wacholder, Dorairaj Prabhakaran, Aleyamma A Mathew

**Affiliations:** 1 National Cancer Institute, National Institutes of Health, Department of Health and Human Services, Rockville, MD, USA; 2 Centre for Chronic Disease Control, New Delhi, India; 3 Sekhsaria Institute for Public Health, Navi Mumbai, India; 4 Westat, Rockville, MD, USA; 5 Regional Cancer Center, Trivandrum, Kerala, India; 6 All India Institute of Medical Sciences, New Delhi, India; 7 Steno Diabetes Center, Gentofte, Denmark; 8 Information Management Services, Silver Spring, MD, USA; 9 United Nations World Food Program, Rome, Italy

## Abstract

**Background:**

India's population exhibits diverse dietary habits and chronic disease patterns. Nutritional epidemiologic studies in India are primarily of cross-sectional or case-control design and subject to biases, including differential recall of past diet. The aim of this feasibility study was to evaluate whether a diet-focused cohort study of cancer could be established in India, providing insight into potentially unique diet and lifestyle exposures.

**Methods:**

Field staff contacted 7,064 households within three regions of India (New Delhi, Mumbai, and Trivandrum) and found 4,671 eligible adults aged 35-69 years. Participants completed interviewer-administered questionnaires (demographic, diet history, physical activity, medical/reproductive history, tobacco/alcohol use, and occupational history), and staff collected biological samples (blood, urine, and toenail clippings), anthropometric measurements (weight, standing and sitting height; waist, hip, and thigh circumference; triceps, sub-scapula and supra-patella skin fold), and blood pressure measurements.

**Results:**

Eighty-eight percent of eligible subjects completed all questionnaires and 67% provided biological samples. Unique protein sources by region were fish in Trivandrum, dairy in New Delhi, and pulses (legumes) in Mumbai. Consumption of meat, alcohol, fast food, and soft drinks was scarce in all three regions. A large percentage of the participants were centrally obese and had elevated blood glucose levels. New Delhi participants were also the least physically active and had elevated lipids levels, suggesting a high prevalence of metabolic syndrome.

**Conclusions:**

A high percentage of participants complied with study procedures including biological sample collection. Epidemiologic expertise and sufficient infrastructure exists at these three sites in India to successfully carry out a modest sized population-based study; however, we identified some potential problems in conducting a cohort study, such as limited number of facilities to handle biological samples.

## Background

According to the World Health Organization, cancer deaths in India are expected to increase 158% by the year 2020. Although overall actual cancer incidence rates are lower in India than in North America and Europe, the rise in cancer-related deaths is likely to present a significant burden to the already overwhelmed health systems. Unique environmental exposures, as well as the genetic variation among people in India, can provide valuable new information on factors that contribute to cancer risk or protect against it [1-3]. Population heterogeneity by region, socio-economic status, and religion provide the opportunity to evaluate a wide range and variety of dietary exposures not easily studied elsewhere, including unique oils, grains, vegetables, and legumes [3,4]. Additionally, spices and chilies in Indian foods contain numerous potential chemopreventive agents yet to be comprehensively examined in epidemiologic studies [5-10].

Epidemiologic studies of diet and cancer in India are primarily of cross-sectional or case-control design and subject to biases, including differential recall of past diet [11-17]. Seminal observations in Japanese migrants and other Asian-Americans have motivated epidemiologic research on unique dietary components related to cancer risk, such as soya products [3,18]. Similarly a prospective cohort study in India could provide insight into the complex relationships between dietary exposures and cancer, as well as provide clues for possible mechanisms [19-21]. Thus, establishing a cohort in India could have a major public health impact for global recommendations by shedding light on cancer risk factors, such as obesity, physical activity, reproductive health, and environmental and occupational exposures [1,22-25]. Early findings would be immediately useful to develop preventive strategies in the at-risk Asian-Indian (AI) and South-Asian populations living throughout the world, where diet and cancer incidence patterns remain unique [3,26-32].

Before establishing a population-based prospective cohort of diet and cancer risk in India, evaluation of specific aspects of the study population, infrastructure, and environment are required. We believe a successful "state-of-the-art" cohort in India would include the following constructs: a wide-range or variability in diet and lifestyle exposures to evaluate associations with cancer risk; the ability to assess exposures using the most current and population-specific methodologies; the capacity to collect biological samples prior to cancer onset and treatment (a particularly important issue in India where many cancer cases present at late stages); and a cost-effective and sustainable, long-term design. Furthermore, although India is a rapidly developing country, the infrastructure necessary for such a study may not be adequate and needs to be evaluated. We also saw a need to assess the potential limitations inhibiting both follow-up and the collection of accurate and unbiased endpoints for a prospective cohort in India [described in [33]].

To evaluate the feasibility of a future diet and cancer cohort, we conducted a multi-center pilot study across three diverse regions of India with population-based cancer registries: New Delhi, Mumbai, and Trivandrum. As opposed to seeking a "random sample", we aimed to capture India's unique heterogeneity in diet and lifestyle exposures to facilitate future epidemiologic investigations of cancer risk. Thus, to further maximize the variability in exposures of interest, namely diet, we recruited diverse religious groups known to follow varied patterns of diet and lifestyle practices; and targeted both urban and rural areas in Trivandrum. In New Delhi and Trivandrum, we also evaluated the feasibility and acceptance of biological sample collection. Similar to established prospective cohorts in developed countries [34-37], we targeted India's large "middle class" population, which was believed to be more stable and accessible than other class extremes; thus, insuring the longevity and long-term follow-up potential of the cohort. The rapidly growing and evolving middle class in India encompasses a wide-range of ethnicities, education-levels and occupations, as well as varied access to resources, such as nutrition and healthcare. In this population, we evaluated different recruitment techniques; collected detailed information on diet, physical activity, and medical history; conducted anthropometric measurements and medical examinations; and collected biospecimens. Herein, we outline the study design and recruitment procedures, provide the response rates for study assessments, and present initial findings of the regional variation in diet, physical activity, anthropometry, and metabolic markers from the India Health Study (IHS).

## Methods

The IHS was conducted between December 2006 and July 2008 in three regions of India: New Delhi in the north (All India Institute of Medical Sciences and Centre for Chronic Disease Control); Mumbai in the west (Healis-Sekhsaria Institute for Public Health); and Trivandrum in the south (Regional Cancer Center). These centers were selected to capture regional variability in diet and lifestyle and to utilize cancer registries meeting the International Agency for Research on Cancer standards [38].

**Figure 1:**
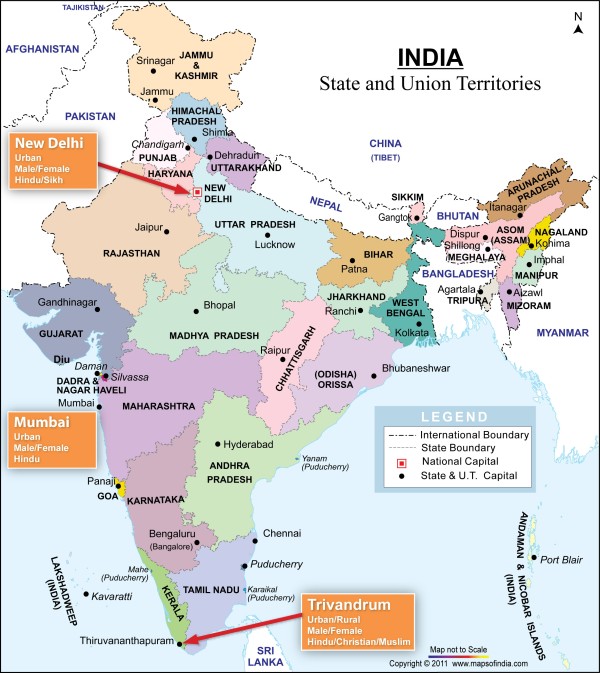
**India Heath Study Centers with the sampling categories**.

Sampling in the IHS was stratified by gender, religion, and type of residence (urban/rural) (Figure 1) and designed to estimate the mean and range of various foods and nutrients within each strata with the following precision: mean intake of nutrients would lie within 5% of the true intake value with 95% confidence and mean intake of foods would lie within 10 percent of the true value with 95% confidence [39,40]. To obtain our desired precision for dietary data and satisfy requirements for estimating the participation rate, approximately 200 households per stratum were required.

Participants were eligible if they were aged 35-69 years old, resided in the study area for a minimum period of one year (to minimize participant migration), had no prior history of cancer or cardiovascular event, could speak English or the primary regional language, had no physical ailments that prevented them from fully participating in the study, and were willing to provide biological samples. Females could not be pregnant. We recruited an approximately equal numbers of subjects for each five-year age category, and one male and one female per household for equal gender distribution and cost efficiency.

Human ethics committees from each study center and the Special Studies Institutional Review Board of the United States National Cancer Institute reviewed and approved the study protocol prior to study commencement. Due to the nature of the international collaboration, the Indian Health Ministry Screening Committee for projects involving foreign assistance and/or collaboration, which is part of the Indian Council of Medical Research reporting to the Government of India, also reviewed and approved the study. We obtained written informed consent from all participants.

**Figure 2:**
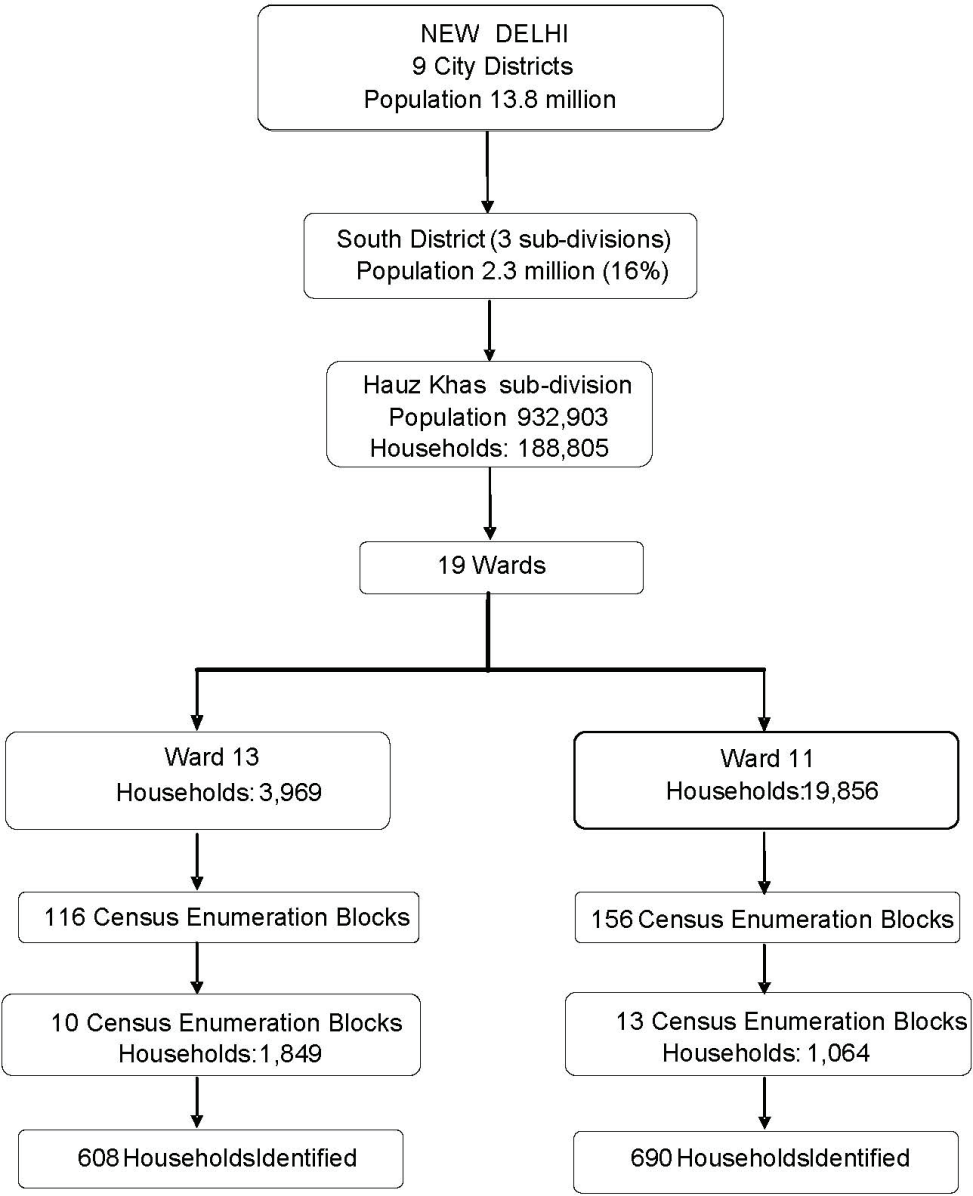
**New Delhi sample selection**.

**New Delhi**, India's capital, is a metropolitan city spread over 1,483 km^2^. The IHS was conducted in the South District, the second largest district with 2.3 million residents (16% of New Delhi's total population), covering 16.7% of the city's total area [41]. Of the three subdivisions in this district, Hauz Khas was randomly selected and of the 19 wards, Wards 13 and 11 were randomly chosen. Within these wards, a total of 23 census enumeration blocks were randomly selected for sampling households (Figure2).

We initially attempted to identify households using the 2001 census information, but the houses listed in the census did not match with the existing properties; therefore, we selected an equal number of houses from each lane of a census enumeration block (contains three to five lanes). Of the 1,298 households identified, we successfully interviewed 626 households.

**Mumbai **(formerly Bombay), the densely populated capital of the state of Maharashtra is divided into suburban districts and the island city, which comprises 15.9% of the greater metropolitan area (76.8 km^2^) with 3.3 million people [41].

**Figure 3:**
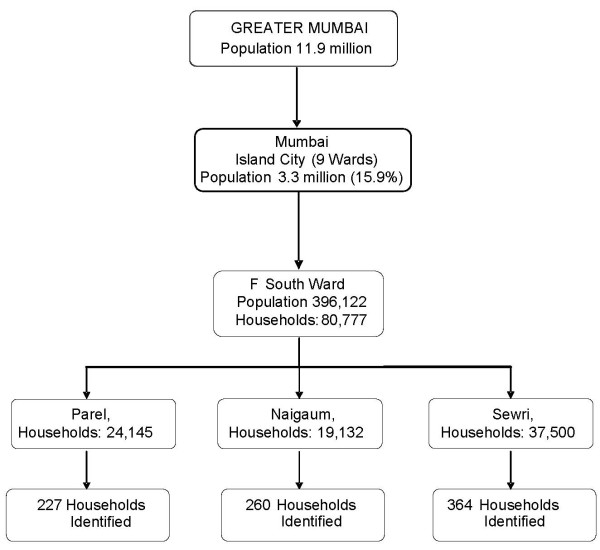
**Mumbai sample selection**.

IHS participants were recruited from an ongoing study of mortality, the Mumbai Cohort Study (39, 40) in three representative areas (Parel, Naigaum, Sewri) from Ward F-South (Figure 3). If the cohort member from a selected household had died, moved outside the study area, or was not eligible for the IHS, then a new eligible person, who may or may not have been a cohort member, was recruited from the same or neighboring household. Of the 851 households identified, 687 households were successfully interviewed.

**Figure 4:**
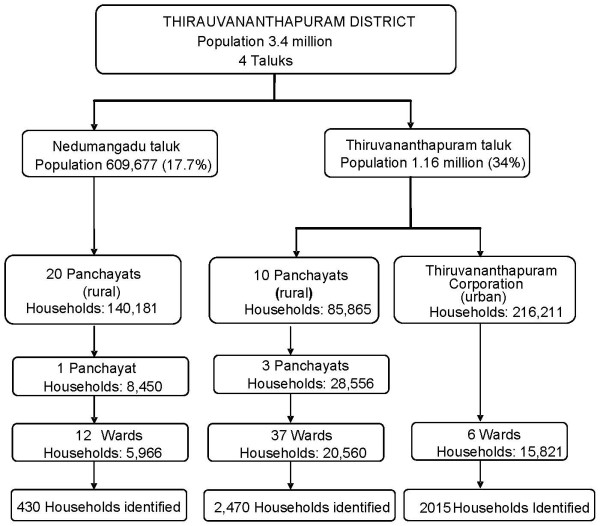
**Trivandrum sample selection**.

**Trivandrum **district (or Thiruvananthapuram), the capital of the state of Kerala, is located on the south-west coast of India, and spans 2,192 km^2^. Trivandrum's 3.4 million people live in four taluks or sub-divisions, which are split into urban areas (34%) and rural panchayats, village councils (76%) [41]. We recruited participants from six urban and 49 rural wards in two taluks (Figure 4).

The wards were selected to maximize the number of Hindu, Christian, and Muslim participants as a proxy for dietary practices. Wards are divided into three or four polling stations and households were identified with voter lists from 2006 that contained the name of the head of household, address, and the number of adult individuals in the household. Of the 4,915 households identified, 1,720 households were successfully interviewed (925 urban, 795 rural).

Before the study recruitment began, the three centers used multiple approaches to introduce the study aims and objectives to the communities. Trained field staff, along with the principal investigators, talked to community and religious leaders, and/or held public meetings in communal areas. In order to standardize the study protocol and implementation, as well as develop a highly-trained and professional staff, we conducted two intensive, week-long protocol development and training sessions. We also instituted a quality control component where principal investigators of the study sites would pay unannounced visits to the field. The community-based mobile clinics or camps, where participants underwent medical examinations and provided biological samples, were staffed by medical doctors and nurses. This was useful to bring attention and credibility to the study's recruitment efforts, as well as to achieve compliance for the different components.

### Study visits

Once households were identified, field interviewers visited the homes to determine eligibility, provide information, and schedule a visit. During the first in-home morning visit, the interviewer administered demographic, residential history, physical activity, tobacco and alcohol use, and occupational history questionnaires, as well as a computerized diet history questionnaire (Table 1). Participants usually completed all questionnaires within one hour. In Mumbai, anthropometric measurements were also completed during the first visit. Interviewers in New Delhi and Trivandrum scheduled a second visit and left biospecimen containers with directions for collecting a first morning urine sample and toenail clipping. Biological samples were not collected in Mumbai as the study center did not have laboratory or storage facilities.

**Table 1:** Field visits and details on questionnaires

Visit	Forms	Details
**Pre-visit** ***Screening***	Household Form	Dwelling type, household members
	Eligibility Screener	Date of birth, gender, language, religion, eligibility information (pregnant, heart attack < 12 months, cancer, hemophilia, willingness to provide blood sample), contact information
	Non-response Form	Reasons for refusal
**Visit 1** ***Questionnaires after signing consent form***	Demographic	Education, mother tongue, marital status, cooking fuel, household appliances, income
	Food Preparer	Household information on use of 19 spices, chilies (dried and green), coconut, garlic, onions, 13 cooking oils
	Residential History	Time at current residence, reason for moving to current residence, contact information in case the participant moves
	Physical Activity	Vigorous activity, moderate activity, walking, sitting during the past 7 days
	Tobacco Use	Type of tobacco smoked/chewed, length of time using product, quantity used
	Alcohol Use	Type of alcohol consumed, the length of time using the product, quantity consumed
	Occupational History	Current occupation, five most recent occupations lasting 6 months or longer, time at each job.
**Visit 2** ***Medical history Physical examination***	Medical History	Information on health care provider, medical conditions, medication, family history
	Reproductive History	Female - menses, sexual activity, pregnancies, contraceptives, hormone therapy Male - sexual activity, contraceptives
	Biospecimen Collection	15 ml fasting blood, first morning urine sample, toe nail clippings
	Anthropometric Measures	Standing and sitting height, weight, blood pressure, waist, hip and thigh circumference, triceps and scapula skinfold

Diet was assessed using a computer-based, interviewer-administered, meal-based comprehensive diet assessment tool known as the New Interactive Nutrition Assistant-Diet in India Study of Health (NINA-DISH) [42,43]. This software was developed for the IHS by modifying software developed by Novo Nordisk Pharma India (Bangalore, India). The diet history (DH) component included three sections: a set of defined questions similar to a food-frequency questionnaire [44], an open-ended section for each mealtime to collect additional unique regional foods, and a food preparer questionnaire (amount and type of oils, spices, onion, garlic, chilies, and coconuts purchased per household). A subset of participants completed four 24-hour dietary recalls providing information on all foods consumed during the day.

All participants completed the validated, short-form of the International Physical Activity Questionnaire (IPAQ) about total time spent in physical activity for recreation, occupation, household work, and transportation in the last 7 days [45-47]. Total weekly physical activity (metabolic equivalents of task (MET-hr/wk) was calculated as the weighted sum of the reported time spent at each intensity using a MET value specific to each category (walking: 3.3 METs; moderate: 4 METs; vigorous: 7 METs).

### Biological sample collection and processing

The second visit was conducted either in mobile clinics within the participant's neighborhood or at the individual's home between six and eight in the morning. The participants completed medical history and reproductive questionnaires, and provided 15 ml of blood, 100 ml of first morning urine sample, and toenail clippings from all toes. As an incentive, we offered to provide the participants with the results from the blood analyses. Blood pressure and anthropometric (weight, standing and sitting height; waist, hip, and thigh circumference; and triceps, sub-scapula and supra-patella skin fold) measurements were also taken. Biological samples were transported to the laboratory in coolers within three-hours of collection. Laboratory technicians processed the samples into fractions as soon as they reached the laboratory (i.e., plasma, serum, blood clot, buffy coat, red blood cells) and stored them in equal aliquots at -80° Celsius. Toenail clippings and Guthrie cards with blood spots were stored in a dry environment at ambient temperature.

Fasting glucose levels were determined with the glucose oxidase/peroxidase method [48] (New Delhi: Randox Laboratories Ltd., Antrim, UK; Trivandrum: Span Diagnostics Ltd., Surat, India). In New Delhi only (all reagents from Randox Laboratories Ltd., Antrim, UK), lipid profiles were analyzed using the following methods: total cholesterol by cholesterol oxidase/p-aminophenazone method, triglyceride by glycerolphosphatase oxidase-peroxidase aminophenazone method, and HDL by precipitation method using phosphotungstate/magnesium-precipitation of apolipoprotein B containing lipoproteins followed by estimation of cholesterol in supernatant by enzymatic method. LDL was estimated using the Friedwald formula [49].

### Follow up projections

We calculated the expected numbers of cancer cases over five years per 100,000 people for the eight most common cancers at each of the four IHS location (i.e., Mumbai, New Delhi, Trivandrum rural and urban) by gender (i.e., total cohort of 800,000). For incidence, we used truncated crude rates (35-69 years) and age-specific rates (35-39, 40-44, 45-49, 50-54, 55-59, 60-64 and 65-69) from the Trivandrum Cancer Registry (2005-2006) [50] and the National Cancer Registry Program (2001-2004) for Mumbai and New Delhi [51].

## Results

**Figure 5:**
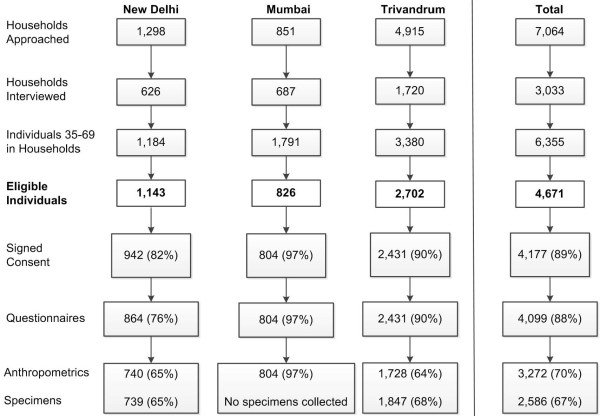
**Individual center and total response rates**.

Of the 6,355 adults between ages 35-69 identified, 4,671 met the eligibility criteria. Of the eligible participants, 89% (4,177/4,671) agreed to participate in the studies by signing the consent form. We calculated the response rates using two denominators: all eligible participants (n = 4,671) and participants who provided informed consent (n = 4,177; Figure 5). Of the 4,671 eligible participants, 88% (4,099/4,671) completed all questionnaires, 70% (3,272/4,671) provided anthropometric measurements, and 67% (2,586/3,845) provided all biological samples (New Delhi and Trivandrum only). Of the 4,177 individuals that agreed to participate in the study, 98% (4,099/4,177) provided all questionnaire information, 78% (3,272/4,177) provided anthropometric measurements, and 77% (2,586/3,373) provided all biological samples (New Delhi and Trivandrum only).

Of the study participants, 78% (739/942) in New Delhi and 76% (1,847/2,431) in Trivandrum, provided both blood and urine. Both types of biological samples were provided by a higher proportion of females (83%), as compared to males (71%) and adherence also differed somewhat by religious group (71% of Christians; 81% of Hindus; and 76% of Muslims; data presented in text only). The proportion of individuals who complied with anthropometric measurements also differed by region (New Delhi, 86%; Mumbai, 95%; and Trivandrum, 77%), gender (87% of females and 78% of males), and religion (73% of Christians; 87% of Hindus; and 76% of Muslims; data presented in text only).

The mean age of the 3,908 IHS participants who completed the demographic and diet history questionnaires was 47, 50, and 49 years in New Delhi, Mumbai, and Trivandrum, respectively (Table 2). Approximately 48% of the total study population was male. New Delhi had the highest proportion of participants with a college degree. Across all regions, men had higher levels of education than women. The majority of the population (> 80%) was married; thus, household income did not differ greatly by gender. History of tobacco use varied by region and gender; 36%, 15%, and 50% of men in New Delhi, Mumbai, and Trivandrum, respectively, reported ever having smoked. Smokeless tobacco was primarily used in Mumbai (36% of men, 52% of women), as compared to New Delhi (6% of men, 1% of women) and Trivandrum (7% of men, 4% of women). The majority of households owned televisions and telephones, while hot water heaters, computers, cars, and washing machines were primarily reported in New Delhi. In 83% of the women, menarche began between the ages of 12 to 15, while 14% were aged 16 years or older (described in text only). Less than 2% of the women had never been pregnant, while 32% had more than 4 pregnancies. Parity did not differ substantially by religion, but women with less than a primary school education were more likely to have 4 or more pregnancies (42%), as compared to women with at least a primary school education (16%; described in text only).

**Table 2:** Selected characteristics of study population by center

Characteristic	New Delhi	Mumbai	Trivandrum
Total (n = 3908)*	n = 839*	n = 768*	n = 2,301*
**Age distribution (years), %**			
Under 40	32	16	21
40-49	31	31	33
50-59	23	31	29
Over 60	14	22	17
**Male, %**	46	48	50
**Religion, %**			
Hindu	77	58	34
Muslim	3	< 1	34
Christian	< 1	6	32
Sikh	18	< 1	0
Buddist	0	35	0
Other	2	< 1	0
**Urban**	100	100	48
**Household monthly income (Indian rupees), %**			
< 5,000	6	36	69
5,000-10,000	18	45	27
> 10,000	76	19	4
**Highest education attained, %**			
Illiterate	6	7	3
Literate, No formal education	6	1	1
Primary school	7	6	13
Middle school	15	24	28
Secondary school	27	56	46
Graduate/University	31	5	7
Post-graduate	8	< 1	3
**Married, %**	94	81	93
**Family members in household**			
(median, 10% and 90%)	5 (3, 6)	5 (3, 8)	4 (2, 6)
**Ever smoked, %**			
Male	36	15	50
Female	2	< 1	1
**Ever other tobacco, %**			
Male	6	36	7
Female	1	52	4
**Types of cooking fuel used, %**			
Firewood	1	0	80
Gas	100	96	78
Kerosene	< 1	39	23
**Household items, %**			
Bicycle	28	6	27
Cable	92	89	61
Car	24	1	8
Computer	18	11	8
Hot water geyser	55	3	1
Phone	95	87	73
Refrigerator	87	78	54
Scooter	63	15	36
Indoor toilet	90	35	55
Television	99	96	89
Washing machine	78	34	12

*3,908 participants had complete demographic and diet history questionnaire information

Missing data excluded from estimates

Food group intake varied widely across the three regions (Table 3). Cereals were widely consumed, but the type varied by region. Participants in Trivandrum frequently consumed fermented rice and rarely consumed wheat products. Compared to relatively low intakes in other regions, unique dietary components by region were fish in Trivandrum, dairy in New Delhi, and pulses (legumes) in Mumbai. Consumption of meat, alcohol, fast food, and soft drinks was scarce in all regions. Fruit and sweet snack intake was also sparse relative to high vegetable consumption. There was also substantial variation in food additives, such as, spices, allium vegetables (e.g., onions, garlic), and cooking oils across the three regions [data not shown; described in [42]]

**Table 3:** Regional distribution of selected food group intake collected in the Diet History Questionnaire

	New Delhi (n = 839)		Mumbai (n = 768)		Trivandrum (n = 2301)	
	%	Median**	%	Median**	%	Median**
Food	≥ 1/month*	(10%, 90%)	≥ 1/month	(10%, 90%)	≥1/month	(10%, 90%)
**Total Cereals**						
Total rice products	97.8	3.5 (1.0, 9.0)	98.2	14.8 (7.2, 18.0)	100.0	19.0 (13.0, 22.5)
Fermented rice	< 1	0 (0, 0)	41.9	0 (0, 2.0)	96.9	4.0 (2.0, 7.0)
Total wheat products	100.0	21.0 (14.5, 28.0)	99.5	21.0 (12.0, 30.0)	80.3	2.0 (0, 8.5)
Refined wheat	64.7	1.00 (0, 6.0)	77.3	3.50 (0, 11.0)	16.2	0 (0, 2.0)
**Vegetables**	99.7	16.3 (6.0, 28.5)	99.9	20.0 (12.0, 44.0)	99.7	15.3 (7.5, 27.0)
Cruciferous	61.1	0.5 (0, 2.2)	84.5	2.0 (0, 4.0)	78.0	1.0 (0, 3.0)
Potato/starchy	98.8	4.5 (1.2, 10.4)	94.9	3.5 (1.0, 8.0)	92.2	1.5 (0.2, 5.0)
**Fruits**	83.5	1.8 (0, 8.0.0)	77.6	3.2 (0, 9.2)	83.8	2.0 (0, 8.0)
Banana	38.7	0 (0, 2.0)	68.8	1.6 (0, 4.0)	82.5	2.0 (0, 7.0)
**Total Pulses**	99.4	6.5 (2.0, 12.0)	99.6	16.0 (9.0, 22.5)	97.7	4.0 (1.8, 7.2)
Pulses with skin	89.0	2.0 (0, 6.2)	97.3	8.0 (3.8, 12.0)	87.0	1.0 (0, 3.0)
**Total Meat**	48.4	0 (0, 3.0)	85.8	2.0 (0, 4.0)	80.5	1.0 (0, 3.5)
Red meat	45.6	0 (0, 1.5)	69.1	0.5 (0, 2.0)	69.4	0.5 (0, 2.0)
**Fish**	20.8	0 (0, 0.5)	84.8	5.0 (0, 9.0)	98.4	14.0 (6.1, 21.0)
Dried fish	< 1	0 (0, 0)	56.0	0.5 (0, 2.0)	36.6	0 (0, 1.0)
**Total Dairy**	98.0	8.0 (1.8, 18.5)	57.9	0.8 (0, 7.0)	72.7	1.0 (0, 7.0)
Curd/Yogurt	96.4	5.0 (1.2, 14.2)	51.2	0.3 (0, 3.0)	70.4	1.0 (0, 4.0)
**Sweet snacks**	83.8	1.0 (0, 2.5)	61.2	0.5 (0, 2.5)	61.8	0.4 (0, 2.6)
**Savory snacks**	78.9	1.2 (0, 4.8)	52.9	0.5 (0, 8.8)	86.3	2.5 (0, 7.8)
**Pickled food**	68.7	1.0 (0, 7.0)	51.6	0.9 (0, 2.0)	72.6	1.3 (0, 7.0)
**Chutneys**	65.6	1.0 (0, 5.0)	65.9	1.0 (0, 4.0)	93.5	2.0 (0.8, 3.0)

*Percent of foods consumed more than once a month.

** Median frequency of intake/week (10th and 90th percentile).

Women had a higher mean body mass index (BMI) than men across all regions (Table 4), but the overall anthropometric distribution did not differ substantially by region or religion (data not shown). When defined by a lower cut-point specific to Asian populations (BMI≥25 kg/m^2^) [52] the prevalence of obesity was close to 50%, while 80% were centrally obese based on the waist-to-hip ratio (WHR). New Delhi participants had the highest BMI and waist circumference and the lowest total physical activity. Across all regions, higher educational attainment was associated with lower levels of physical activity and higher BMI (data not shown). Trivandrum had the highest prevalence (61% in urban areas and 46% in rural areas) of impaired fasting glucose (≥100 mg/dl) [53,54] and over 25% of the participants in the urban areas could be classified as diabetic (≥ 126 mg/dl), while only 18% self-reported a medical history of diabetes. In New Delhi men and women, respectively, 53% and 48% had total cholesterol levels over 200 mg/dL, and 40% and 30% had LDL to HDL ratios above 4.5.

**Table 4:** Anthropometric measures, physical activity, and blood markers by study center and gender

	New Delhi		Mumbai		Trivandrum			
					Rural		Urban	
	Female	Male	Female	Male	Female	Male	Female	Male
	(n = 451)	(n = 388)	(n = 399)	(n = 369)	(n = 595)	(n = 596)	(n = 550)	(n = 560)
Weight (kg)	62.8 (49, 79)	69.9 (54, 88)	58.2 (44, 73)	63.0 (50, 81)	60.5 (46, 73)	66.2 (50, 77)	62.8 (47, 78)	65.9 (50, 80)
								
Height (cm)	155 (147, 161)	167 (160, 176)	150 (143, 158)	164 (156, 173)	152 (143, 161)	164 (155, 172)	153 (145, 163)	164 (155, 172)
								
Physical Activity (MET-hr/wk)	30 (12, 100)	26 (8, 102)	175 (15, 342)	139 (12, 400)	127 (51, 263)	135 (34, 331)	154 (36, 263)	118 (17, 330)
								
BMI (kg/m^2)^	26.4 (21, 33)	25.0 (20, 30)	25.3 (20, 32)	23.3 (19, 28)	25.0 (20, 31)	23.8 (19, 27)	26.2 (21, 33)	24.5 (19, 29)
								
Waist (cm)	92.3 (79, 110)	95.1 (82, 108)	86.7 (72, 103)	88.4 (75, 103)	83.9 (73, 98)	85.0 (74, 98)	88 (74, 102)	86.0 (75, 99)
								
WHR	0.96 (0.83, 1.03)	0.98 (0.92, 1.05)	0.90 (0.81, 0.98)	0.97 (0.89, 1.05)	0.93 (0.83, 1.02)	0.97 (0.89, 1.05)	0.93 (0.84, 1.03)	0.98 (0.92, 1.06)
								
WHR High*, %	85	95	80	88	87	88	87	92
WTR High^**^, %	82	79	-	-	55	62	57	68
								
FPG, mg/dL	103.0 (86, 140)	103.0 (87, 157)	-	-	92.0 (62, 168)	96.0 (62, 171)	100.0 (71,175)	100.0 (70, 171)
≥100 mg/dL, %	61	60	-	-	44	46	63	59
≥126 mg/dL, %	15	16			17	17	28	25
								
Diabetes, self-report, %	11	13	10	12	21	25	17	18

Abbreviations: BMI, body mass index; WHR, waist-to-hip ratio; WTR, waist-to-thigh ratio; FPG, fasting plasma glucose; Median (10%, 90%) unless otherwise specified as percent.

*WHR ≥ 0.9 (m) ≥ 0.85 (f); **WTR ≥ 2

Based on existing cancer registry data in India [50,51] a cohort of 800,000 with 5-years of follow-up would accrue a total of approximately 5,000 cases of the 8 most common cancers (Table 5).

**Table 5:** Expected number of cases of the most frequent cancers (based on cancer registry data) in a potential cohort of 800,000 people (100,000 per gender and region)

			Trivandrum		
Site	New Delhi	Mumbai	Rural	Urban	Total
**Male**					
Oral cavity/pharynx	155	165	255	165	740
Esophagus	40	45	50	35	170
Stomach	25	30	85	65	205
Colorectal	40	40	50	120	250
Lung	110	60	225	185	580
Prostate	35	25	35	80	175
	**405**	**365**	**700**	**650**	**2,120**
No. of cancers in males (shown above) by age categories:					
Under 40	27	16	15	20	78
40-49	86	76	120	140	422
50-59	140	126	340	195	801
Above 60	152	147	225	295	819
					
**Female**					
Oral cavity/pharynx	50	75	120	45	290
Esophagus	30	35	10	5	80
Stomach	15	15	15	10	55
Colorectal	30	35	55	50	170
Lung	30	30	40	35	135
Breast	315	295	460	370	1440
Cervix uteri	190	145	140	85	560
	**660**	**630**	**840**	**600**	**2,730**
No. of cancers in females (shown above) by age categories:					
Under 40	72	57	100	40	269
40-49	210	192	260	185	847
50-59	209	203	275	205	892
Above 60	169	178	205	170	722

## Discussion

We conducted the IHS to evaluate whether a complex multi-center, population-based diet and cancer cohort with multiple questionnaires and biological samples could be implemented in India. The IHS demonstrated that it is feasible to recruit a large number of adults from different regions of India with an ample response rate. Questionnaires, physical examinations, and biological samples (in New Delhi and Trivandrum) were successfully obtained. However, our study also identified areas that require attention before a cohort study could be established in these populations.

We discovered that a large percentage of participants agreeing to take part in an epidemiologic study would complete all components of the study. These findings are noteworthy, as the conventional wisdom at the time the IHS was initiated held that many of the participants would not agree to provide blood or urine samples or to undergo physical examinations. Our results should encourage future researchers in India to collect biological samples, so that results are comparable to other international studies. However, it is important to note that the recruitment was restricted to individuals who agreed to provide biological samples, which could be an important strategy for future studies. Even though this method could lead to recruitment bias, the internal validity of the study results would not be compromised if there is minimal differential loss to follow-up.

The reputation of the local institutes and the professional attitude of the interviewers were critical to obtaining a high participation rate, as individuals often changed their minds regarding enrollment once staff mentioned the name of the affiliated organization. We also used extensive community outreach (talking to village leaders, open meetings in communities) to provide study information. We observed that having uniformed doctors and nurses on the field team engendered trust from participants, especially for biological sample collection and physical examination; and we found that receiving test results (blood pressure, glucose, cholesterol, and hemoglobin) was a cost-effective incentive for enrollment and participation.

We did, however, encounter challenges with collection, transport, and storage of biological samples. In Mumbai, we could not collect biological samples as adequate laboratory and storage facilities were not available. We found that weather was an important factor in the other regions where we collected biological samples. During the summer, it was difficult to keep samples cold, and in Trivandrum, dry ice necessary for shipping samples was not available. In New Delhi, home visits during the winter months presented logistical difficulties for field staff. During monsoon season, visits often could not be completed. Transferring the biological samples for analysis within three hours was achievable in our study, as most of the collection sites were relatively close to the processing laboratory. A similar strategy may be possible in a full-scale study by establishing temporary processing centers and/or mobile clinics. For the purposes of this study, we also found that we needed to set up reliable short- and long-term biological sample storage. We encountered various problems such as the availability of dry ice for shipment of samples to the central repository, electrical outages, diesel fuel shortages for backup generators, and technical support delays during freezer breakdowns. Some of these problems could be ameliorated with the use of liquid nitrogen freezers. Other large cohorts have had to address many of these issues and may provide creative solutions [55].

The diet history data revealed substantial differences in regional food consumption [43]. For the full study population, the diets encompass a wide variety of foods not consumed regularly in other populations (e.g., fermented rice, pulses or lentils, and vegetables). Variability in dietary components added during cooking also provided novel information that may help evaluate some preventive or adverse aspects of the Indian diet [42]. For example, while coconut oil and ghee contain high levels of saturated fat that could be detrimental, mustard seed oils may have beneficial properties [56]. Future studies that assess these food items could provide valuable information on the chemopreventive properties of spices (e.g., curcumin), allium vegetables, and chilies in relation to chronic disease etiology [5,7,57,58].

We also observed variation in lifestyle factors, such as tobacco use and physical activity. Tobacco use varied greatly between the genders, as in India smoking is not considered a socially acceptable behavior for women. In the limited number of women that reported tobacco use, we found that they were more likely to use smokeless products. The regional differences in tobacco use, such as the preference for smokeless tobacco in Mumbai compared to smoking in Trivandrum (namely in men), may be a reflection of the varied distribution of education and/or socioeconomic status across the regions. Physical activity levels were lowest in New Delhi and throughout the regions physical activity appeared to be inversely related to education and income level, perhaps reflecting occupation-related activity and/or use of labor-saving devices.

The IHS population appears to fit the "AI-phenotype" [59], a model of metabolically obese, normal-weight individuals [60]. To account for differences in body composition across different ethnicities, the World Health Organization provides modified cut points for BMI that may be more applicable to Indian and other Asian populations [52]. When using these criteria, just under half of participants in the IHS participants were obese. Compared with Caucasian populations, AIs typically develop metabolic syndromes at lower BMIs, and for any given waist circumference, have increased visceral fat and insulin resistance, but thinner extremities [61-63]. Measures approximating the proportion of visceral versus subcutaneous fat deposition (WHR and WTR) may be particularly useful in this population. Using these measures, we found that compared to Western populations more individuals in the IHS were centrally obese, a predictor of type 2 diabetes [64,65]. In New Delhi and Trivandrum, fasting blood analysis revealed that many IHS participants were pre-diabetic and dyslipidemic; and thus, at high risk for diabetes and cardiovascular disease [62,66].

It is important to note that our sampling scheme and recruitment methods may have decreased the external validity of our study population, as we encountered several challenges during recruitment. Study personnel had limited access to individuals of higher socioeconomic status [67,68], as they tended to live in gated communities. Furthermore, we did not include individuals in temporary housing or of very low socioeconomic status from urban areas, due to potential issues with very limited dietary variability (i.e., only cereal) and major challenges with follow-up, particularly for medical outcomes. Since younger, healthier males were more likely to work outside of their homes during the study visit time-frame we may also have higher proportions of women and older individuals. We recognize that an alternative sampling method for selecting individuals from households [69] may be useful in the future to capture a more representative sample. We also realize that while the generalizability or external validity may be an issue, the internal validity of study results would not be compromised if the loss to follow-up was non-differential.

## Conclusions

From this feasibility study, we conclude that there is epidemiologic expertise and sufficient infrastructure at these three sites in India to successfully carry out a population-based study of a modest size. However, our current cancer incidence projections suggest that a prospective cohort study investigating the relationship of diet to cancer outcomes would need to recruit close to one million participants to accrue approximately 5,000 cases over 5-years of follow-up, requiring a greater level of manpower and expertise. Epidemiologists, statisticians, study and field managers, computer programmers, dieticians/nutritionists, medical staff, trained interviewers, and laboratory technicians would be needed in greater numbers to expand this feasibility study in its current form. In addition to fully utilizing local expertise, it may also be necessary to expand public health training programs in these areas. Based on our experiences, it would also be crucial to have personnel who understand the cultural and administrative issues at each study site. Biospecimen storage presents a greater logistic challenge. Field stations for processing samples close to the collection sites or mobile clinics may alleviate some of the immediate issues. However, stable biospecimen storage at the study centers, as well as long-term storage at a central repository would also need to be established. Finally, developing a validated computerized system for all study questionnaires should be considered to ease data collection and management, as we found that dietary information could be collected on laptops even in rural settings.

## Abbreviations

AI: Asian Indians; BMI: body mass index; CEB: community enumeration block; DH: diet history; HT: hypertension, HIS: India Health Study; IPAQ: International Physical Activity Questionnaire; PA: physical activity; WHR: waist-to-hip ratio; WTR: waist-to-thigh ratio

## Competing interests

The authors declare that they have no competing interests.

## Authors' contributions

The author responsibilities were as follows - RS designed, initiated, and secured study funding, developed analytic strategy, interpreted the results, and drafted the manuscript; CRD, LMF contributed to the development of the analytic strategy, interpretation of results, and drafting of the manuscript; SW, KSR, NC, TR participated in the study design and drafting of the manuscript; ND, HS, PSG implemented the overall site-specific study protocol, such as recruitment, field-work, data collection; BIG provided statistical support; SY, KGM, MJM developed protocols and oversaw data management and analyses; LR oversaw the biological collection protocol and measured biomarkers; KK developed the dietary assessment instrument; PCG, DP, AAM were principal investigators of the three centers and involved in the concept and design of the India Health Study. All authors read and approved the final manuscript.

## Pre-publication history

The pre-publication history for this paper can be accessed here:

http://www.biomedcentral.com/1471-2458/11/405/prepub
